# Gastrointestinal helminths of intensively managed poultry in Kwara Central, Kwara State, Nigeria: Its diversity, prevalence, intensity, and risk factors

**DOI:** 10.14202/vetworld.2019.389-396

**Published:** 2019-03-14

**Authors:** Shola David Ola-Fadunsin, Patricia Isioma Uwabujo, Idiat Modupe Sanda, Isau Aremu Ganiyu, Karimat Hussain, Musa Rabiu, Nusirat Elelu, Musbau Olayinka Alayande

**Affiliations:** 1 Department of Veterinary Parasitology and Entomology, Faculty of Veterinary Medicine, University of Ilorin, P.M.B. 1515 Ilorin, Kwara State, Nigeria; 2 Department of Veterinary Public Health and Preventive Medicine, Faculty of Veterinary Medicine, University of Ilorin, P.M.B. 1515 Ilorin, Kwara State, Nigeria; 3 Department of Veterinary Parasitology and Entomology, Faculty of Veterinary Medicine, Usmanu Danfodiyo University, P.M.B. 2346 Sokoto, Sokoto State, Nigeria

**Keywords:** epidemiology, gastrointestinal helminths, Kwara State, Nigeria, poultry

## Abstract

**Aim:**

Helminth infections inflict negatively on the production and well-being of animals including poultry. This study was carried out to determine the prevalence, species diversity, intensity, and risk factors associated with the gastrointestinal helminths of intensively raised poultry in Kwara Central senatorial district of Kwara State.

**Materials and Methods:**

Fecal samples were collected from 502 poultry species from 15 farms. The samples were subjected to floatation and the formalin-ethyl acetate concentration techniques of examination. The intensity of infections was determined using McMaster counting technique.

**Results:**

Seven helminth species were detected with *Heterakis* *gallinarum* (10.2%) and *Ascaridia galli* (6.0%) been the most prevalent, while *Capillaria* species was the least prevalent (0.8%). Physiological status, bird type, production purpose, farm age (years), presence of other animals in the farm, flock size (birds), farm size (acres), housing type, farm type, frequency of anthelmintic use, distance to waste area (meters), level of biosecurity, and frequency of cleaning the pen were the risk factors significantly (p<0.05) associated with the presence of helminth infections.

**Conclusion:**

This study shows that helminth infections are endemic in the study area, as 66.7% of the sampled farms were infected with one or more helminth species. Findings from this study provide information that will assist in improving the poultry sector in Kwara State, Nigeria in general, for better production and profitability.

## Introduction

Poultry are domesticated birds kept by man for the purpose of obtaining meat, eggs, sometimes feathers, and as a means of livelihood. They include birds such as chicken, duck, goose, and turkey [1]. It is one of the most important sources of protein and farm manure, for man and so their importance cannot be overemphasized [1,2]. Poultry production has increased constantly throughout the world over the past decades, and according to the Food and Agriculture Organization [3], around 75% of a total of 15 billion chickens is found in the developing countries [4]. In Nigeria, poultry is an important component of the livestock subsector with a total population of over 200 million [5,6]. This sector has developed to the level of commercial enterprise which provides employment, income, and animal protein for urban and rural dwellers as well as manure for crop production [6]. It is an important instrument for alleviating problems associated with poverty in Nigeria and other developing countries (in terms of food security and malnutrition) [6].

The diseases condition caused by helminth infections is known as helminthosis. This condition has been considered as an important problem of poultry in Nigeria and other parts of the world [7-9]. Helminth parasites have been incriminated as a major cause of ill-health and loss of productivity through decreased feed conversion ratio, reduced weight gain and weight loss in broilers, poor egg lay in layers, and mortalities. Helminthoses are also associated with catarrh, diarrhea, intestinal obstruction, loss of appetite, anemia, weakness, paralysis, and poor feathering in birds [1,2,7,9,10]. Helminth parasites of poultry are commonly divided into three main groups; nematodes, cestodes, and trematodes. Nematodes constitute the most important group of helminth parasites of poultry, both in number of species and the extent of damage they cause. Few numbers of cestodes and trematodes are known to parasitize poultry [7,11,12]. The prevalence and intensity of helminth infections may be influenced by several factors, such as climatic conditions (temperature and humidity) which may alter the population dynamics of the parasites, resulting in dramatic changes in the prevalence and intensity of helminthic infections [12]. Many insects that may act as vectors for helminths are also favored by high temperatures and to some extent humidity [1,12].

To the best of our knowledge, there is no report on gastrointestinal helminths of poultry in this part of the country. This study is, therefore, carried out to determine the prevalence, species diversity, intensity, and risk factors associated with the gastrointestinal helminths of intensively raised poultry with the aim of providing information on this subject matter that will help in better profitability in the poultry sector in the state and country.

## Materials and Methods

### Ethical approval

All applicable international, national, and/or institutional guidelines for the collection of fecal samples from avian species were correctly followed.

### Informed consent

Informed consent was obtained from all participants.

### Description of study location

**Figure-1:**
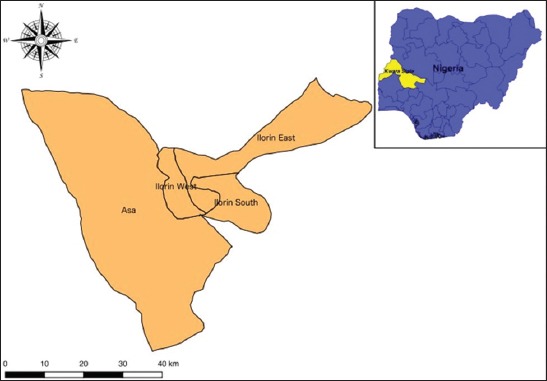
Map of Kwara Central (the study location). The insert map shows Kwara State within Nigeria (Designed using QGIS Version 2.6.1).

This study was conducted in Kwara Central senatorial district of Kwara State. Kwara Central lies almost in the middle of Nigeria, and it is one of the major linkages between the northern and southern part of the country. Kwara Central comprises four local government areas (Asa, Ilorin East, Ilorin South and Ilorin West). Kwara State is located between latitude 8°05N and 10°15N and longitude 2° 73E and 6°13E. It is located in the middle belt (North Central) within the forest-savanna region of Nigeria (Figure-1). The state is bordered in the west by Benin Republic, in the east by Kogi State, and the south by Oyo, Osun, and Ekiti States. Kwara State population is about 3 million people, and it covers a total area of 34,500 km^2^ comprising rainforest in the south and wooded savannah in the larger part of the state. It has 16 local government areas. The state has two seasons, the dry and wet season, with heavier rainfall in September and October. The state has a mean annual rainfall of between 112.8 cm and 146.9 cm and an average annual temperature ranging from 22.1°C to 33.3°C. It records a mean relative humidity of 49.6% [5,13].

### Study design and sampling

A total of 502 fecal samples were collected from 15 poultry farms located within Kwara Central (Figure-1), comprising layers, broilers, and mixed species farms. The farms were visited between December 2017 and May 2018 following permission by the farm owners. Birds were monitored and individually freshly voided fecal samples were immediately collected from the ground and placed into well-labeled sterile sample bottles and put in a cool box. The samples were immediately transported to the Parasitology Laboratory of the Faculty of Veterinary Medicine, University of Ilorin, Nigeria, for further processing.

### Processing of fecal sample

Fecal samples were processed using the simple floatation and the formalin-ethyl acetate concentration techniques. The floatation technique was carried out as described by Soulsby [14]. Briefly, 2 g of each fecal sample was mixed with quantity of saturated sodium chloride solution and filtered (using a sieve) into a glass test tube. Afterward, the mixture was filled to the brim (forming a meniscus) with the saturated sodium chloride solution, and a clean coverslip was gently placed on top of the test tube, thereby avoiding spillage. The coverslip was left for about 20 min; afterward, the coverslip (having the harvested eggs) was placed on a clean glass slide and examined with the light microscope using the 10× and 40× objective lenses.

The formalin-ethyl acetate concentration technique was carried out as described by Cheesbrough [15]. Briefly, about 2 g of each feces was dissolved in 10% formalin and sieved into a plastic test tube to the 7 ml mark and allowed to stand for a few minutes. 3 ml of ethyl acetate was added. The tube was closed, vigorously shaken by hand for 1 min, and centrifuged at 3000 rpm for 5 min. The debris plug was loosened, and the top three layers were discarded. Iodine stain preparation was made with the sediment, and the entire sediment was examined on a clean glass slide and covered with a clean coverslip. The covered slides were examined using 10× and 40× objective lenses.

### Fecal egg counts

Samples that were positive for helminth eggs were subjected to the McMaster counting technique as described by Soulsby [14], with modifications to make use of a smaller volume of saturated sodium chloride solution. Briefly, 2 g of feces was properly dissolved in 12 ml of saturated sodium chloride solutions (as against 60 ml). The solution was then filtered through a tea sieve into a beaker. The filtrate was pipetted into the two chambers of the McMaster slide. Afterward, it was allowed to stand for 30 s. Finally, it was examined using the 10× objective lens. All the eggs seen within the ruled areas were counted. The eggs per gram (EPG) was estimated using the formulae below:


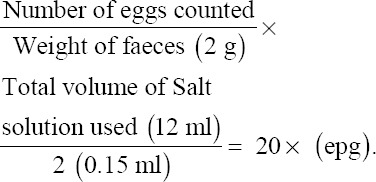


### Identification of helminth eggs

The eggs from the processing methods described above were identified using the helminthological keys as described by Soulsby [14] and Taylor *et al*. [16].

### Determination of positivity

Samples that were positive in one or both of the tests carried out were considered positive for the helminth(s) detected.

### Determination of prevalence (%) and mean intensity (EPG)

The total prevalence (%) of each helminth species was calculated as the total number of poultry infected with each helminth detected divided by the total number of poultry sampled (502), while the farm-based prevalence (%) was calculated as the total number of poultry infected with a particular helminth detected in each farm divided by the total number of poultry sampled in that farm. The mean intensity (EPG) was calculated by summing the total EPG from all infected birds having a particular helminth species in a farm divided by the number of birds infected with the particular helminth parasite in that farm.

### Questionnaire design and administration

A well-structured, interviewer-administered questionnaire containing open-ended and closed-ended (dichotomous or multiple choices) questions was designed to obtain information on individual bird that sample was collected from, the poultry farm demography, environmental and management factors, and biosecurity. A respondent was someone who was actively involved in the daily activities of the farm and was not necessarily the farm owner.

### Statistical analysis

The data were statistically analyzed using the “Microsoft Excel 2010 and SPSS-Version 22.0” (SPSS Inc., Chicago). Descriptive statistics were conducted to estimate the prevalence using percentages in tables. The univariate analysis (Chi-square) test and odds ratios (ORs) with 95% confidence interval (CI) were used to determine the association between each risk factor and the presence and absence of helminth parasites. The ORs were calculated with respect to a reference category as indicated in the respective tables. p<0.05 was considered statistically significant for all analyses.

## Results

### Overall prevalence (%) of helminth parasites

**Figure-2:**
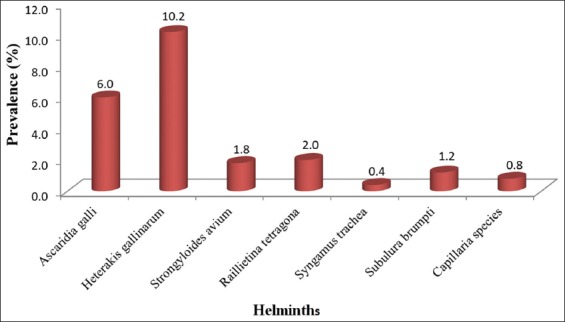
Prevalence (%) of gastrointestinal helminths of intensively managed poultry in Kwara Central, Kwara State.

Seven different species of helminth (six nematodes and one cestode) were detected from our study. Of the 502 birds sampled, 10.2% (51/502; 95% CI=7.7-13.0) were infected with *Heterakis* *gallinarum*, while 0.4% (2/502; 95% CI=0.1-1.3) were infected with *Syngamus trachea*. The prevalence of the other helminths ranged between 0.8% and 6.0% (Figure-2).

### Prevalence (%) of helminth parasites coinfection among poultry

Of the sampled birds, 84 (16.7%) were infected with one helminth or the other. 66 of them were infected with one helminth species representing 13.2% (95% CI=10.4-16.3). Of this category, *H*. *gallinarum* was the most prevalent (33/502; 6.6%; 95% CI=4.6-9.0), while *Subulura brumpti* was the least prevalent (2/502; 0.4%; 95% CI=0.1-1.3). *Ascaridia galli* + *H*. *gallinarum* combination was the most prevalent in the two helminth parasites coinfection representing 1.6% of the sampled population. Two birds had three helminth parasites coinfection representing 0.4% (2/502; 95% CI=0.1-1.3) of the sampled population. Four of the sampled birds were infected with four helminth parasites at the same time, with the infection been *A. galli* + *H. gallinarum* + *S. brumpti* + *Capillaria* species (2/502; 0.4%) and *H*. *gallinarum* + S. trachea + *S. brumpti* + *Capillaria* species (2/502; 0.4%) (Table-1).

**Table-1:** Prevalence (%) of gastrointestinal helminths coinfection among intensively managed poultry in Kwara Central, Kwara State.

Gastrointestinal helminth (s)	Number positive (%)	95% CI
One helminth infection	66 (13.2)	10.4; 16.3
*Ascaridia galli*	18 (3.6)	2.2; 5.5
*Heterakis gallinarum*	33 (6.6)	4.6; 9.0
*Strongyloides avium*	5 (1.0)	0.4; 2.2
*Raillietina tetragona*	8 (1.6)	0.7; 3.0
*Subulura brumpti*	2 (0.4)	0.1; 1.3
Two helminths infection	12 (2.4)	1.3; 4.0
*Ascaridia galli+Heterakis gallinarum*	8 (1.6)	0.7; 3.0
*Heterakis gallinarum+Raillietina tetragona*	2 (0.4)	0.1; 1.3
*Heterakis gallinarum+Strongyloides avium*	2 (0.4)	0.1; 1.3
Three helminths infection	2 (0.4)	0.1; 1.3
*Ascaridia galli*+H*eterakis* *gallinarum*+S*trongyloides avium*	2 (0.4)	0.1; 1.3
Four helminths infection	4 (0.8)	0.3; 1.9
*Ascaridia galli+Heterakis gallinarum+Subulura brumpti+Capillaria* species	2 (0.4)	0.1; 1.3
*Heterakis gallinarum+Syngamus trachea+Subulura brumpti+Capillaria* species	2 (0.4)	0.1; 1.3

CI=Confidence interval

### Prevalence (%) of helminth parasites in the different poultry farms

The farm prevalence of helminth infections is presented in Table-2. Of the 15 farms visited, five were free from helminth infections representing 33.3%. One farm had birds that were infected with six of the seven helminth species detected, while other farms were infected with between one to three helminth species. *H*. *gallinarum* and *A. galli* had the widest spread, been detected in eight and seven farms, respectively, while the other helminth species were detected in one or two farms. In general, individual helminth prevalence within farms ranged between 6.3% (*A. galli* in farm 3) and 56.7% (*H*. *gallinarum* in farm 8).

**Table-2:** The prevalence (%) of gastrointestinal helminth parasites from the different poultry farms in Kwara Central, Kwara State.

Farms	n	Gastrointestinal helminths (number infected [prevalence %])							

		*Ascaridia galli*	*Heterakis* *gallinarum*	*Strongyloides avium*	*Raillietina* *tetragona*	*Syngamus trachea*	*Subulura brumpti*	*Capillaria* species	Number of helminths
1	80	nf	nf	nf	nf	nf	nf	nf	nf
2	30	4 (13.3)	nf	nf	nf	nf	nf	nf	1
3	32	2 (6.3)	4 (12.5)	6 (18.8)	nf	nf	nf	nf	3
4	30	nf	4 (13.3)	nf	nf	nf	nf	nf	1
5	30	2 (6.7)	9 (30.0)	3 (10.0)	nf	2 (6.7)	6 (20.0)	4 (13.3)	6
6	30	3 (10.0)	5 (16.7)	nf	nf	nf	nf	nf	2
7	30	2 (6.7)	3 (10.0)	nf	5 (16.7)	nf	nf	nf	3
8	30	13 (43.3)	17 (56.7)	nf	nf	nf	nf	nf	2
9	33	nf	nf	nf	nf	nf	nf	nf	nf
10	30	nf	nf	nf	nf	nf	nf	nf	nf
11	30	4 (13.3)	nf	nf	nf	nf	nf	nf	1
12	27	nf	nf	nf	nf	nf	nf	nf	nf
13	30	nf	nf	nf	nf	nf	nf	nf	nf
14	30	nf	5 (16.7)	nf	5 (16.7)	nf	nf	nf	2
15	30	nf	4 (13.3)	nf	nf	nf	nf	nf	1

nf=Not found

### Mean intensity of infections (EPG of feces) of helminth parasites in the different poultry farms

The highest mean intensity of infections was recorded in *H*. *gallinarum* (981.0) and closely followed by *A. galli* (751.4). *Strongyloides avium*, *Raillietina* *tetragona*, *S. trachea*, *S. brumpti*, and *Capillaria* species had a mean intensity of 360.0, 90.0, 60.0, 60.0, and 30.0, respectively. The individual mean intensity of infections within farms was highest in farm 4 with *H*. *gallinarum* recording 290.0 (±127.0) and lowest in farm 5 with *Capillaria* species recording 30.0 (±11.5) (Table-3).

**Table-3:** The mean (±SD) intensity of gastrointestinal helminths (EPG of feces) of intensively managed poultry in individual farms in Kwara Central, Kwara State.

Farms	Gastrointestinal helminths (mean [±SD] intensity of infections [EPG])							Total

	*Ascaridia galli*	*Heterakis* *gallinarum*	*Strongyloides avium*	*Raillietina* *tetragona*	*Syngamus trachea*	*Subulura brumpti*	*Capillaria* species	
1	nf	nf	nf	nf	nf	nf	nf	nf
2	160.0 (8.2)	nf	nf	nf	nf	nf	nf	160.0
3	100.0 (14.14)	160.0 (23.1)	180.0 (99.6)	nf	nf	nf	nf	440.0
4	nf	290.0 (127.0)	nf	nf	nf	nf	nf	290.0
5	160.0 (23.1)	96.0 (63.1)	180.0 (28.3)	nf	60.0 (28.3)	60.0 (35.7)	30.0 (11.5)	586.0
6	70.0 (11.5)	80.0 (46.2)	nf	nf	nf	nf	nf	150.0
7	60.0 (14.1)	70.0 (11.5)	nf	50.0 (11.5)	nf	nf	nf	180.0
8	151.4 (55.3)	145.0 (64.3)	nf	nf	nf	nf	nf	296.4
9	nf	nf	nf	nf	nf	nf	nf	nf
10	nf	nf	nf	nf	nf	nf	nf	nf
11	50.0 (11.5)	nf	nf	nf	nf	nf	nf	50.0
12	nf	nf	nf	nf	nf	nf	nf	nf
13	nf	nf	nf	nf	nf	nf	nf	nf
14	nf	60.0 (16.3)	nf	40.0 (17.9)	nf	nf	nf	100.0
15	nf	80.0 (69.3)	nf	nf	nf	nf	nf	80.0
Total	751.4	981.0	360.0	90.0	60.0	60.0	30.0	2332.4

nf=Not found, SD=Standard deviation, EPG=Eggs per gram

### Risk factors associated with helminth parasitic infection

The association between bird type and the occurrence of helminth infections was statistically significant (p<0.05). Layers (18.5%) were 6.6 times more likely to be infected with helminth parasites compared to turkeys (3.3%). Spent layers (80.0%) were significantly (p<0.05) more prone to helminth infections compared to productive (16.1%) and unproductive (14.3%) birds. Poultry birds raised for the purpose of meat were twice less prone to the infection than those raised for egg purpose. Farms that are <15 years of the establishment are of higher risk of infection compared to farms older than 15 years. The presence of other animal types in the farm increases the chances of helminth infections (OR=2.0). The risk of helminth infections decreases with the increase in flock size, farm size, and distance to waste area. Birds raised in the deep litter had a lower risk (0.4) of infection compared to those raised in a battery cage. Birds raised in farms in the presence of other bird types were of higher risk (3.3) of infection compared to birds raised in farms with single bird species. Frequency of anthelmintic use, level of biosecurity, and frequency of cleaning the pen were other risk factors significantly associated (p<0.05) with the occurrence of helminth infections (Table-4).

**Table-4:** Risk factors associated with helminth infections among intensively managed poultry in Kwara Central, Kwara State.

Variables	Helminth+ve (%)	Helminth –ve (%)	OR (95% CI)	p-value
Age (weeks)				
Chick (0-8)	1 (3.85)	25 (96.15)	0.19 (0.01, 1.07)	0.06
Grower (>8-16)	13 (19.70)	53 (80.30)	1.19 (0.60, 2.27)	0.59
Adult (>16) a	70 (17.07)	340 (82.93)	1.00	
Sex				
Male	4 (7.84)	47 (92.16)	0.40 (0.12, 1.05)	0.06
Female^a^	80 (17.74)	371 (82.26)	1.00	
Bird type				
Layers	74 (18.50)	326 (81.50)	6.6 (1.2, 13.6)	0.02^b^
Broilers	9 (12.50)	63 (87.50)	4.1 (0.63, 9.53)	0.17
Turkey^a^	1 (3.33)	29 (96.67)	1.00	
Physiological status				
Unproductive	26 (14.29)	156 (85.71)	0.04 (0.01, 0.20)	<0.01^b^
Productive	50 (16.13)	260 (83.87)	0.05 (0.01, 0.22)	<0.01^b^
Spent layers^a^	8 (80.00)	2 (20.00)	1.00	
Production purpose				
Meat	10 (9.80)	92 (90.20)	0.48 (0.23, 0.94)	0.03^b^
Egg^a^	74 (18.50)	326 (81.50)	1.00	
Farm age (years)				
<5	32 (21.05)	120 (78.95)	28.85 (5.36, 60.34)	<0.01^b^
>5-10	28 (18.67)	122 (81.33)	24.83 (4.58, 52.4)	<0.01^b^
>10-15	23 (25.56)	67 (74.44)	36.92 (6.64, 78.53)	<0.01^b^
>15-20^a^	1 (0.91)	109 (99.09)	1.00	
Presence of other animals in the farm				
Yes	68 (19.32)	284 (80.68)	2.00 (1.14, 3.68)	0.02^b^
No^a^	16 (10.67)	134 (89.33)	1.00	
Flock size (birds)				
<1000	68 (22.67)	232 (77.33)	24.23 (4.64, 49.87)	<0.01^b^
1000-2000	15 (13.56)	103 (86.44)	11.99 (2.08, 25.98)	<0.01^b^
>2000^a^	1 (1.19)	83 (98.81)	1.00	
Farm size (acres)				
<5	79 (23.80)	253 (76.20)	24.58 (4.72, 50.50)	<0.01^b^
5-10	4 (4.44)	86 (95.56)	3.65 (0.45, 9.99)	0.26
>10^a^	1 (1.25)	79 (98.75)	1.00	
Housing type				
Deep litter	8 (8.33)	88 (91.67)	0.40 (0.17, 0.82)	0.01^b^
Battery cage^a^	76 (18.72)	330 (81.28)	1.00	
Farm type				
Multiple bird species	68 (22.52)	234 (77.48)	3.34 (1.90, 6.11)	<0.01^b^
Single bird species^a^	16 (8.00)	184 (92.00)	1.00	
Frequency of anthelmintic use				
Every 2 months	22 (32.35)	46 (67.65)	4.41 (1.70, 12.82)	<0.01^b^
Every 3 months	52 (19.12)	220 (80.88)	2.20 (0.94, 5.91)	0.07
Occasionally	4 (4.00)	96 (96.00)	0.39 (0.09, 1.49)	0.17
No at all^a^	6 (9.68)	56 (90.32)	1.00	
Distance to waste area (meters)				
<250	11 (17.74)	51 (82.26)	5.74 (0.91, 13.09)	0.07
250-500	72 (17.48)	340 (82.52)	5.71 (1.05, 11.99)	0.04^b^
>500^a^	1 (3.57)	27 (96.43)	1.00	
Level of biosecurity (%)				
0-25	64 (19.39)	266 (80.61)	1.83 (1.08, 3.20)	0.03^b^
25-50^a^	20 (11.63)	152 (88.37)	1.00	
Frequency of cleaning the pen				
Twice a week	35 (13.16)	231 (86.84)	8.30 (1.54, 17.41)	0.01^b^
Weekly	48 (26.67)	132 (73.33)	19.86 (3.70, 41.47)	<0.01^b^
Occasionally^a^	1 (1.79)	55 (98.21)	1.00	

a Reference category.

b Significant. OR=Odds ratio, CI=Confidence interval

## Discussion

Little or nothing is known about gastrointestinal helminth parasites of intensively managed poultry in Kwara Central as it pertains to its diversity, prevalence, intensity, and risk factors. The overall prevalence of 16.7% reported in this study is much lower than previous studies conducted in Nigeria: 42.5% [1], 81.0% [17], and 100.0% [7] and outside Nigeria: 84.6% in Bangladesh [4] and 91.9% in Iran [18]. The reason for the low prevalence recorded may be associated with the fact that the birds recruited for this study were intensively managed where they get better treatments in terms of biosecurity, hygiene, feeding, and appropriate preventive medical programs and general management. Higher helminth infections have been reported in extensively and semi-intensively raised birds compared to intensively raise domestic chickens [10].

The result of this study showed that seven species of helminths affect intensively managed poultry in the study area. Similar to our finding, Adang *et al*. [17] reported seven gastrointestinal helminth species in domestic chickens in Gombe State, Nigeria. Contrary to our finding, Yoriyo *et al*. [19] reported eight gastrointestinal helminth species among chickens in Bauchi State, six helminth species in Abuja [11], five species in Akure, Ondo State [10], and four species in Sokoto State [20]. Outside Nigeria, 16 helminth species have been reported among chickens in South Africa [8], eight helminth species in India [21], seven species in Trinidad [9], six species in Bangladesh [18], four species in Iran [22], and three helminth species in Poland [23]. The differences in the number of helminth species detected in this study vis-à-vis those of other studies could be attributed to environmental and climatic differences. Our finding shows that there are diverse species of gastrointestinal helminths affecting poultry in Kwara Central of Kwara State.

This study reported that *H*. *gallinarum* and *A. galli* were the predominant helminth species, with *S. avium*, *R*. *tetragona*, S. trachea, *S. brumpti*, and *Capillaria* species been of lesser prevalence. This is comparable with that recorded by other authors [7,10,24], who recorded *H*. *gallinarum* and *A. galli* as the most prevalent helminth species of poultry in their studies conducted in Nigeria and Iran [25], respectively. Low prevalence of *S. avium*, *R*. *tetragona*, S. trachea, *S. brumpti*, and *Capillaria* species has also been reported in Nigeria [1,11,24,26]. The high prevalence of *H*. *gallinarum* and *A. galli* may be associated with the peculiarity in their life cycle as the eggs of both helminths can remain viable in the soil for several months [16], whereby prolonging the contamination time in the environment as birds constantly pick up viable eggs from the droppings that contaminate the environment as they feed, and this also predisposes them to high prevalence and heavy parasite burden.

The helminths coinfection observed in this study is a common phenomenon in most poultry species [9,17,27]. This may be associated with the fact that some helminth infections require intermediate/paratenic host (e.g., earthworm) which can harbor and transmit more than one helminth species at a time [16].

The presence of helminth parasites in 66.7% of the visited farms shows that helminth infections are endemic among farms in the study area. Helminth has been known to cause reduced weight gain and weight loss in broilers, poor egg lay in layers, and mortalities [2,10], thereby resulting in production loss. The widespread of *H*. *gallinarum* and *A. galli* among farms confirms that these helminth species are the most endemic in the area and most parts of Nigeria as documented by previous researchers [1,11].

The mean intensity of infections showed not to be alarming as no helminth count was above 300 EPG. This low count may be attributed to the management system (intensive) in which the birds were raised. Nevertheless, this does not translate to optimum production in poultry as low intensity of helminth infections may also cause problems in a flock.

Higher prevalence of helminth infections was recorded in layers and broilers compared to turkey. This may not be readily explained, although prevalence of 68.3% has been reported in turkeys [26] compared to 88.4% in layers [10] with both works done in Nigeria. Spent layers had a higher prevalence of helminth infections compared to unproductive and productive birds. A study conducted in Germany reported that free-range chickens at the end of the laying period (spent layers) had greater intensity and prevalence of infection with *A. galli* and *H*. *gallinarum* compared to hens kept in closed poultry houses (in preparation for production or during production) [28]. This finding may be attributed to the fact that spent layers are not given much care in terms of medications and biosecurity as these birds are merely kept to be sold for meat. Moreover, one cannot rule out the effect of immunosuppression associated with aging which may also be a reason. Birds raised for the purpose of meat production had a lower prevalence of helminth infections compared to birds raised for egg production. Similarly, Afolabi *et al*. [10] reported a significantly lower prevalence of helminth infections in broilers compared to layers in their study carried out in Nigeria.

Birds raised in farms where other animals are present showed to be more prone to helminth infections compared to those raised in farms without other animal species. This is expected as other animals may serve as a vehicle in the transfer of viable helminth eggs into the farm [10,16]. Furthermore, the biosecurity in poultry farms where other animals are present (e.g., dogs) will be seriously compromised. Contrary to the expected outcome, birds raised in the deep litter had a lower prevalence of helminth infections compared to those raised in a battery cage. Bachaya *et al*. [29] and Teni *et al*. [30] reported that birds raised on deep litter were more infected with helminths than those raised in a battery cage. The frequent use of anthelminthic drugs by farms that raise their birds on deep litter may be the reason behind the contrary report between our study and other previous studies. The higher risk of infection seen in birds raised in the presence of other avian species may be associated with cross infections between different bird species.

Interestingly, birds that are occasionally treated with anthelmintics had the lowest prevalence of helminth infections while those that are treated every 2 months were most infected. This report may be attributed to anthelmintics resistance that is associated with its frequent use. Frequent use of anthelmintics increases the resistant population of nematodes [31,32].

Strunz *et al*. [33], Ikpeama *et al*. [34], and Taiwo *et al*. [35] have associated higher prevalence of helminth infections with proximity to waste area and level of sanitation and hygiene. These set of researchers reported that proximity, presence of waste, and poor level of hygiene and sanitation trigger the availability of helminth infections in man and livestock. In line with the report of these aforementioned researchers, we discovered an indirect relationship between the distance of to waste areas and level of biosecurity with the prevalence of helminth infections. Our findings may be attributed to the fact that helminths are known to require warmth, good humidity, and optimum temperature for eggs to hatch and develop to infective stage(s). Studies have shown that poor biosecurity provides favorable conditions for helminth infections in poultry [36,37].

## Conclusion

The findings of this study show that helminth infections are endemic in the study area, with *H*. *gallinarum* and *A. galli* been the most prevalent among the seven species detected. Two-third of the sampled farms was infected with one helminth parasite or the other. There was a low mean intensity of infections, and this will not rule out the economic effect, helminthosis cause on production. A number of factors were significantly associated with the positivity of helminth infections. This study will be essential for policy-making in other to improve poultry production in Kwara State and Nigeria as poultry occupy a pivotal aspect of the national livestock sector.

## Authors’ Contributions

SDO designed the study and was involved in sample collections and laboratory work. He also did the data analysis and drafted the manuscript. PIU was involved in the sampling and in laboratory work. IMS, IAG, KH, MR, and MOA were involved in the laboratory work. NE designed the map of the study location (figure-1) and was involved in sampling. All authors read and approved the final manuscript.
